# Exploring the characteristics of nursing agencies in South Africa

**DOI:** 10.3402/gha.v8.27878

**Published:** 2015-05-11

**Authors:** Omolola I. Olojede, Laetitia C. Rispel

**Affiliations:** Centre for Health Policy & Medical Research Council Health Policy Research Group, School of Public Health, Faculty of Health Sciences, University of the Witwatersrand, Johannesburg, South Africa

**Keywords:** nursing agency, nurses, labour broker, health workforce, South Africa

## Abstract

**Background:**

Nursing agencies are temporary employment service providers or labour brokers that supply nurses to health establishments.

**Objective:**

This study was conducted to determine the characteristics of nursing agencies and their relationship with clients in the health sector.

**Methods:**

During 2011, a cross-sectional national survey of 106 nursing agencies was conducted. After obtaining informed consent, telephone interviews were conducted with a representative of the selected nursing agency using a pretested structured questionnaire. Questions focused on the following: ownership, date of establishment, province of operation, distribution of clients across private and public health facilities; existence of a code of conduct; nature of the contractual relationship between nursing agencies and their clients, and numbers and cadres of nurses contracted. The survey data were analysed using STATA^®^ 12.

**Results:**

Fifty-two nursing agencies participated in the survey, representing a 49% response rate. The study found that 32 nursing agencies (62%) served private-sector clients only, which included private hospitals, homes for elderly people, patients in private homes, and private industry/company clinics, and only four (8%) of the agencies served the public sector only. Twenty-seven percent of nursing agencies provided services to homes for elderly individuals. Nursing agencies were more likely to have contracts with private-sector clients (84%) than with public-sector clients (16%) (*p* = 0.04). Although 98% of nursing agencies reported that they had a code of conduct, the proportion was higher for private-sector clients (73%) compared to public-sector clients (27%). In terms of quality checks and monitoring, 81% of agencies agreed with a statement that they checked the nursing council registration of nurses, 82% agreed with a statement that they requested certified copies of a nurse's qualifications. Only 21% indicated that they conducted reference checks of nurses with their past employers.

**Conclusions:**

Nursing agencies should enhance their quality assurance mechanisms when engaging contracted staff. Overall, the study findings suggest the need for improved governance and management of nursing agencies in South Africa.

The health workforce crisis remains a priority in all countries around the world (1), but is particularly acute in Africa. In South Africa, addressing health workforce challenges is critical in order to achieve health development goals (2). Nurses in South Africa, as elsewhere, make up the largest single group of health-care providers, and their role in attaining quality health care services cannot be over-emphasised (3, 4). They play various roles in the health sector, and they are often the link between communities or patients and health-care facilities (5). However, there are numerous challenges faced by this group of health-care providers. These include, *inter alia*, changes in disease patterns; growing demand for health-care services; an ageing nursing workforce; a shortage of nurses; and an increasing process of casualisation of nursing work, evidenced by practices such as moonlighting (having a second job additional to a primary job) and agency nursing (3, 6–8).

Casualisation, or the employment of workers on a contract part-time basis without the benefits associated with permanent employment, is a global phenomenon (9). Casual employment is done typically through a temporary employment service (TES) agency or labour broker, that employs the casual worker and then contracts the person to a company, organisation, or individual that needs the service (10). Casualisation is a triangular form of employment that includes a third party who is an intermediary between the employee and the employer. Thus there is no formal relationship between the employee and employer, and labour laws do not always protect the employees (10). Figure 1 illustrates the relationship between the casual worker, the TES agency (the labour broker), and the employer.

**Fig. 1:**
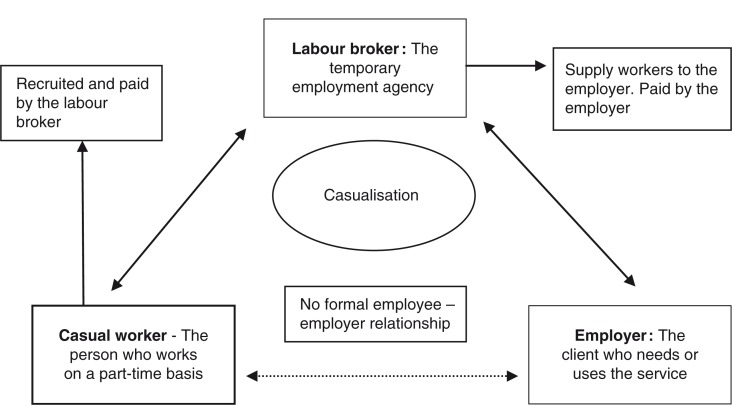
Triangular employment in labour brokering.

This method of employment has also found its way to the health sector, influenced by globalisation of the health workforce, individual preferences for flexibility, and the additional income it offers (11). Developments in the health sector are particularly evident in the employment of nurses through nursing agencies, which play the role of TES agencies or labour brokers (12, 13).

In South Africa, anecdotal evidence suggests that there has been a growth of the nursing agency industry in the past decade. The 1978 Nursing Act (Section 1) defines a nursing agency as ‘a business which supplies registered nurses or midwives or enrolled nurses or nursing auxiliaries to any person, organisation or institution, whether for gain or not and whether in conjunction with any other service rendered by such business or not’ (14).

Much of the literature on nursing agencies comes from Australia, Canada, the United Kingdom, and the United States (11, 15–24). This literature is of limited use in this study, because it tends to focus on agency nurses, rather than on the nursing agency industry. The focus of the literature is on nurses’ motivation for agency employment, management of agency nurses by the hospital administration, the quality of health delivery by agency nurses, and permanent nurses’ relationship with agency nurses (11, 15–24). In South Africa, research has shown that there is widespread utilisation of nursing agencies in the public health sector (6). In the 2009–2010 financial year alone, 1.49 billion South African rands (US$212.64 million) were spent on nursing agencies in this sector (6). There is also extensive utilisation of nursing agencies in the private health sector (25). Notwithstanding the health-care expenditure on nursing agencies, nurses moonlight through commercial nursing agencies. A 2010 cross-sectional survey found that 37.8% of study participants engaged in agency nursing in the year preceding the survey (7).

Little is known about nursing agencies except that they play the role of labour brokers in the health sector. At the time of the study, there were heated debates in South Africa on the future of labour brokers and on the negative impact of nursing agencies on the public health sector (26–28). In light of limited empirical information and a proposed ban on labour brokers in South Africa, this study was conducted to determine the characteristics of nursing agencies and their relationship with clients in the health sector and to explore possible health policy implications of the findings.

## Methods

During 2011, a cross-sectional national survey of nursing agencies was conducted. Ethical clearance for the study was obtained from the University of the Witwatersrand's Human Research Ethics Committee (Medical). Standard ethical procedures were adhered to. These included a detailed information sheet, informed consent, and voluntary participation.

The sampling frame consisted of all registered nursing agencies on the 2010 database of the South African Nursing Council (SANC). The latter is a regulatory authority established in terms of the Nursing Act that governs the nursing and midwifery professions in South Africa (14). Historically, commercial nursing agencies were required by law to register with the SANC (14). At the time of the study, the SANC database presented the best available information, as neither the Department of Health nor the Department of Labour had any database or consolidated information on nursing agencies.

Nursing agencies that were members of the Association of Nursing Agencies in South Africa (ANASA), a voluntary umbrella body set up to represent the industry, were excluded because a separate study focused on ANASA members. Agencies that had closed and were no longer in operation were also excluded. In cases where agencies had multiple branches, only one branch was selected to represent the agency.

**Fig. 2:**
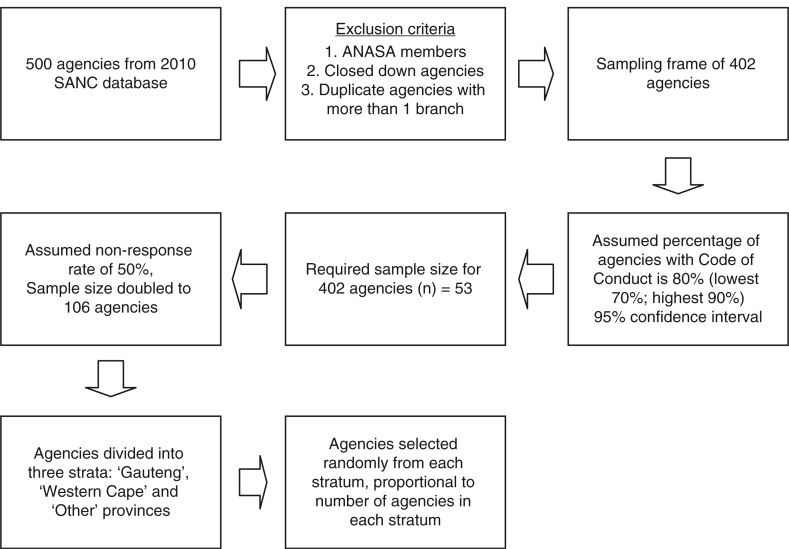
Sampling approach and calculation of sample size.

Previous information indicated that the majority of nursing agencies were located in the urban provinces of Gauteng and the Western Cape. The agencies were grouped into three strata, namely ‘Gauteng’, ‘Western Cape’, and ‘Others’. A stratified random sample of agencies was then selected from each stratum, proportional to the number of agencies in each stratum, totalling a sample of 106 nursing agencies. The details of the sampling procedure are shown in Fig. 2.

An introductory call was made to each of the selected agencies to explain the purpose of the study and to invite their participation. The information sheets and consent forms were sent by email or fax to the agencies. Once the agency representative had agreed to participate, the questionnaire was completed over the telephone.

The survey questionnaire was pretested and structured to focus on the following: agency ownership, date of establishment, province(s) of operation, distribution of clients across private and public health facilities, the existence of a code of conduct (typically an agreement between the agency and the client that sets out principles of engagement, ethical conduct, and mechanisms of communication), the nature of the contractual relationship between nursing agencies and their clients, and the number and categories of nurses employed. Using a seven-point Likert scale, the advantages and challenges of agencies were elicited using a series of statements to which a representative of the agency was asked to indicate the extent of agreement or disagreement. A final open-ended question elicited any comments or suggestions about nursing agencies or the health system.

To ensure a high response rate, an average of three follow-up calls on three separate days were made during office hours to each agency, but in some instances up to nine calls were made to an agency.

The data were analysed using STATA^®^ 12. The Likert scale options of strongly agree, agree, and slightly agree were pooled into one group labelled ‘agree’, while strongly disagree, disagree and slightly disagree were grouped as ‘disagree’. A descriptive analysis was conducted that included frequency tabulations of characteristics of nursing agencies and further cross-tabulations of each of these characteristics by province to investigate any statistical associations. The Fisher's exact test or the Chi-square test was used to test associations between variables. All tests were done at a 95% confidence interval. The qualitative information from the response to the open-ended question was analysed using thematic content analysis (29).

## Results

There were 52 nursing agencies that participated in the survey, representing a 49% response rate. Of these, 26% of the nursing agencies were located in Gauteng Province, 9% in the Western Cape, and 15% were from the other seven provinces.

There were four categories of the non-respondents (51%) in the study: 18% of the agencies were not operational or were closed, and this aspect only became known during the actual fieldwork; 16% of agencies did not answer the phone, despite a minimum of three phone calls on three separate days during office hours; 2% of agencies were ineligible because they were members of ANASA, and this only came to light during the survey; and 15% of agencies refused to participate in the study.

### Characteristics of nursing agencies

The overall characteristics of responding nursing agencies are shown in Table 1. The majority of agencies (*n* = 40; 77%) were established between 2000 and 2009. Most of the agencies surveyed were not owned by larger organisations (90%); only five agencies had a parent organisation (10%), and 83% of agencies had only one branch. One agency from Gauteng Province had five branches.

**Table 1:** Characteristics of nursing agencies

Characteristics of nursing agencies	Gauteng		Western Cape		Others		Total		
	
Sample size (*n*)/%	*n*=27	%	*n*=9	%	*n*=16	%	*n*=52	%	p*
Mean number of years in business (SD)	8.2 years (SD = 7.5)								
	Clients of nursing agencies [No. (%)]^a^								
Homes for elderly people	8	30	4	44	2	13	14	27	0.197
Provincial Departments of Health	4	15	3	33	3	19	10	19	0.50
Private Hospital Group 1^b^	4	15	2	22	1	6.3	7	14	0.506
Private Hospital Group 2^b^	5	19	0	–	4	25	9	17	0.277
Private Hospital Group 3^b^	4	15	1	11	4	25	9	17	0.621
Other private hospitals	3	11	0	–	4	25	7	14	0.187
Private patients	11	41	6	67	6	38	23	44	0.322
Private-sector clients only^c^	–	–	–	–	–	–	32	62	
Public-sector clients only^d^	–	–	–	–	–	–	4	8	
No public- or private-sector clients	–	–	–	–	–	–	10	19	
Public- and private-sector clients	–	–	–	–	–	–	6	12	
	Year established [No. (%)]								
≤1964	1	4	0	–	0	–	1	2	0.530
1986–1994	1	4	1	11	1	6	3	6	
1995–1999	4	15	3	33	1	6	8	15	
2000–2009	21	78	5	56	14	88	40	77	
	Branches of agencies [No. (%)]								
1 branch	24	89	7	78	12	75	43	83	0.566
2 branches	2	7	1	11	3	19	6	12	
3 branches	0	–	1	11	1	6	2	4	
5 branches	1	4	0	–	0	–	1	2	
	Ownership of agencies [No. (%)]								
Owned by larger organisations	1	4	2	22	2	13	5	10	0.272

a These were not mutually exclusive.

b There are three large private hospital groups in South Africa – numbers are used here for the sake of anonymity.

c Private-sector clients are combined: private hospitals, homes for elderly people, private home patients, and private industry/company clinics.

d Public-sector clients are combined Provincial Departments of Health.

* *P*-value from Chi-square test of association.

At the time of the survey, 27% of nursing agencies had homes for elderly people as clients, followed by the provincial departments of health (19%). At least 10 agencies supplied casual staff to at least one provincial department of health. A total of 23 (44%) nursing agencies served private patients in their homes. There were no statistical significant differences between the characteristics of the nursing agencies and the province in which they were located (Table 1).

Table 1 also shows that 32 nursing agencies (62%) served private clients only, 6 (12%) agencies served both public and private clients, whereas 10 (19%) nursing agencies served neither public-sector nor private-sector clients and had no clients at the time of the survey or were inactive.

### Staff contracted by nursing agencies

In terms of categories of casual staff, 86% of agencies reported that they contracted professional nurses, 65% contracted enrolled staff nurses, whereas 61% and 67% of agencies contracted nursing assistants and caregivers, respectively. The median number of casual staff (nurses or caregivers) registered with agencies was 50 (IQR = 15–120).

### Relationship between nursing agencies and clients

Overall, 77% indicated that they had formal contracts or agreements with their clients. Nursing agencies were more likely to have contracts with private-sector clients (84%) than with public-sector clients (16%), and this difference was statistically significant (*p=*0.04). Although 98% of nursing agencies reported that they had a code of conduct, the proportion was higher for private-sector clients (73%) than for public-sector clients (27%). However, this difference was not statistically significant. Similarly, the majority of agencies (96%) stated that they had a reporting mechanism for client complaints, and this applied to 72% of private-sector clients, as opposed to 28% of public-sector clients. This client reporting mechanism was primarily verbal, rather than in writing (Table 2).

**Table 2:** Nursing agencies’ reported relationships with their clients

Variable	Private-sector clients^a^ (%)	Public-sector clients^b^ (%)	Total (%)	*P*
Formal contracts with clients	31 (84)	6 (16)	37 (77)	0.04*
Policy to guide supply of nurses	30 (73)	11 (27)	41 (82)	0.57
Existence of code of conduct	35 (73)	13 (27)	48 (98)	0.70
Existence of client complaint reporting mechanism	34 (72)	13 (28)	47(96)	0.53

a Private-sector clients are combined: private hospitals, homes for elderly people, private home patients and private industry/company clinics.

b Public-sector clients are combined provincial departments of health.

* Statistically significant at 0.05 level.

Participating agencies reported that the supply of casual staff to clients is mostly based on a client's demand for the type of staff needed (92%), rather than on the individual nurse's preference (35%). Nursing agencies reported that the median number of nursing staff or caregivers allocated on a daily basis to clients was 15 (IQR = 5–31.5).

In terms of clinical services (closely related to the types of clients), 37% of agencies provided nurses for geriatric care, followed by adult intensive care units (35%) and other services (10%). Other services included HIV testing in some private organisations and occupational health services.

In terms of quality checks and monitoring, 81% of agencies agreed with a statement that they checked the SANC registration of nurses, 82% agreed with a statement that they requested certified copies of a nurse's qualifications. Only 21% indicated that they conducted reference checks of nurses with their past employers.

### Challenges experienced by nursing agencies

Table 3 shows the agencies’ responses to a series of statements in the questionnaire on possible challenges experienced by them.

**Table 3:** Reported challenges experienced by nursing agencies

Reason	% Agreement
There is a shortage of specialised nurses.	94
We find it challenging to recruit nurses.	83
Fixed commission rate.	80
The government is supportive of nursing agencies.	67
Hospitals are willing to partner with agency on nursing training.	44
Hospitals pay their fees on time.	44
Nurses are committed and loyal professionals.	29
Client expectations of nursing agencies are clear.	29
The performance of retired nurses is unsatisfactory.	28
It is easy to communicate with the hospitals.	18

Three themes emerged from the responses to the open-ended question: governance of nursing agencies, client-related issues, and issues related to individual nurses. These topics overlap and are elaborated on below.

Fifteen of the comments related to issues of governance of nursing agencies, with 10 commenting on the perceived lack of support from the SANC when complaints about nurses were submitted or information about their registration was requested. They also commented on the SANC's lack of support for caregivers (lay health workers). Minor comments related to the policy uncertainty in light of the debates at the time regarding the proposed ban on labour brokers, lack of recognition of nursing agencies, and the high membership fees of ANASA, the voluntary association for nursing agencies.

Another set of comments related to the clients of nursing agencies, notably the high competition for clients among the various agencies, especially those owned by the large private hospital groups (*n=*7); lack of nurse orientation in some private hospitals (*n=*1); inability to meet clients’ demand (1); and racial discrimination or bias in selecting some agencies (*n* = 1).

With regard to individual nurses, agencies complained about the unreliability of nurses (*n=*4), the alleged misconduct of nurses (*n=*2), and the quality of care provided by nurses (*n=*1).

## Discussion

This study was done at a time of heated debate in South Africa on the future of labour brokers and on the negative impact of nursing agencies on the public health sector (26–28). The study found that the majority of agencies surveyed (77%) were established between 2000 and 2009. Because we do not have comparative data prior to this period, it is difficult to determine whether this number represents a significant increase in the number of agencies established during this period. One explanation could be that nursing agencies do not last very long, so that by the time of the survey only those that had been established in the past decade were still in business. One the other hand, the establishment of the agencies could be linked to the health workforce challenges experienced in the South African health system. For example, in 1994, there were 251 nurses per 100,000 population, compared to 110 per 100,000 in 2007; hence, fewer nurses were available relative to population size (30). As with all cross-sectional surveys, the temporal sequence between the establishment of a nursing agency and nursing shortages or possible growth in labour force casualisation could not be determined.

The study found that 32 (62%) of the surveyed agencies served private clients only and did not have any public-sector clients. Just over one-quarter (27%) of nursing agencies' clients were homes for elderly people, and geriatric care comprised an important component of the clinical services that these agencies provided. These findings suggest that homes for elderly people and geriatric care could be the niche areas of these smaller nursing agencies. It might be that ANASA members are the main providers of nursing services to large hospitals, particularly in the public sector, and that the smaller agencies could or would not compete with the larger agencies for the patronage of hospitals. As the findings of the ANASA study have not yet been analysed, it is not possible to compare the findings in this study with that of the ANASA study. However, any legislative or policy initiative on nursing agencies would need to take account of the nursing care needs of private homes or those for elderly people.

In this study, all but four nursing agencies (98%) indicated that they had a code of conduct, and 77% of agencies indicated that they had formal contracts with clients. Although this is a requirement of the Basic Conditions of Employment Act (BCEA), which states that labour brokers are to be jointly responsible for an employee (31), it is encouraging that so many nursing agencies reported the existence of a code of conduct. However, nursing agencies with private-sector clients were more likely to report formal contracts with clients (84%), compared to those who had formal contracts with non-private-sector clients (16%). This is of concern, because one would expect similar or greater accountability by public-sector clients for public monies spent on nursing agencies. As was the case with the nursing agency study in Australia (24), this study found that the predominant method that clients used to report complaints to nursing agencies was informal and verbal.

The study found that nurses or caregivers were allocated based on clients’ demands, rather than on the nurse or caregiver's preference or skills. Other studies have found that when nurses are not allocated to clients according to their preferences, the quality of care is compromised (19, 20, 32). This survey found that 81% of agencies agreed with a statement that they checked the SANC registration of nurses and 82% agreed with a statement that they requested certified copies of a nurse's qualifications. Of concern, however, is that almost one-fifth of agencies did not seem to comply with the basic quality checks of checking registration or requesting copies of qualifications. Only one-fifth of agencies (21%) indicated that they conducted reference checks with past employers. Hence this is an area that needs improvement, because the failure of a nursing agency to conduct these basic quality checks could lead to serious negative incidents both for individual clients and for the health system as a whole (33).

The highest reported challenges by agencies were nurse-related, notably shortages of specialised nurses (94%) and recruitment of nurses (83%). This suggests that the country's overall shortage of nurses, especially in specialised areas, also affects nursing agencies (34). This could lead to competition between agencies and health-care facilities for the employment of the limited number of specialised nurses, thus exacerbating the overall health workforce problems in South Africa.

This study has found that a monitoring system for agency nurses was lacking. In theory, a nurse could register and work with more than one agency within a short time period or the registration or qualifications of a nurse might not be checked, thus impacting on quality of care, a finding supported by an Australian study (12). The nursing agencies themselves raised the issue of governance in response to the open-ended question. The study findings point to the need for tighter management and regulation of agencies and improved monitoring and evaluation.

This study has revealed that this group of agencies that are not members of ANASA provided services to homes for elderly people and private homes, similar to the findings of a study done in a UK health district (35). Because 27% of nursing agencies provided services to homes for elderly people, that have remained outside the mainstream debates on labour brokers, these views would need to be incorporated into health policy development.

Despite careful planning and specific steps to minimise bias, which included doubling of the required sample size from 53 agencies to 106 agencies, a major weakness of the study was the low response rate of 49%. However, this response rate is much higher than an Australian study that had a response rate of 23% (24). In this study, non-operational agencies and no response to numerous phone calls accounted for 34% of non-respondents, indicating problems with the SANC database. These database problems arose due to a legislative vacuum, because SANC is no longer responsible for nursing agencies, which were classified as health establishments by the National Health Act (36).

The study did not ask for the reasons why these agencies were not operational, but one reason could be a lack of sustainability of smaller agencies. This is an area for further research. Only 15% of agencies contacted refused to participate, hence it is possible that with an accurate and updated database, the actual response rate would have been much higher. ANASA members were excluded, which limits the generalisability of the study findings to all nursing agencies.

Telephone surveys do not allow anonymity of responses, hence there could be social desirability bias, particularly in the responses to questions about the code of conduct and quality checks on nurses. Some of the questions might have been misunderstood by respondents and the person being interviewed may have had limited knowledge on the agency they worked for. The study was limited by the small sample size, which may explain some of the findings that were not statistically significant. In the analysis, some responses were not mutually exclusive, which limited the type of analysis conducted. Future studies regarding nursing agencies can build on the questionnaire used in this study and refine the questions to ensure that they are mutually exclusive.

Nevertheless, there are many study strengths. The focus is novel, and it is one of the first studies to focus on the nursing agency industry in South Africa. The findings provide a basis for future research on the nursing agency industry and sheds light on the characteristics of the industry. Selecting a stratified random sample, rather than a convenient sample of agencies, is a strength. The study provides unique information on agencies that are not part of ANASA.

There are a number of recommendations that flow from this study. A first step in strengthening the management of nursing agencies should be the development of a comprehensive database of all registered agencies in the country, which should be updated on a yearly basis. In terms of the National Health Act (36), a comprehensive set of regulations on nursing agencies should be developed, covering both the clients and staff contracted by the agencies. The guidelines should draw on best practice in other countries such as the United Kingdom (22, 23), as well the results of this study. The regulations should include a set of quality standards that are part of the national core standards (37).

Nursing agencies are health establishments (36) but are also labour brokers. Hence they face a dilemma of governance as they have to report to the National Department of Health (NDOH), the SANC, and the National Department of Labour (NDOL). There is a need for a consensus document that will outline the roles and responsibilities of the three organisations in regulating the industry. The NDOH could create a special unit to monitor the activities of nursing agencies annually. This special unit could be within the independent Office of Health Standards Compliance and could work together with a responsible unit at the NDOL to enforce labour standards. All agencies should be mandated to provide information to the responsible government institution on a set of core indicators that covers both their clients and the casual staff that they contract.

## Conclusion

The South Africa health system faces numerous problems of recruitment, management, and retention of health-care providers (4). Casualisation of the workforce compounds these challenges, especially in the public health sector. The study findings underscore the need for improved management, governance, and regulation of nursing agencies and enforcement of existing legislation. In the long term, there is a need for open policy debate on the future of nursing agencies to ensure that they meet the needs of the South African health system.
